# Successful Use of Combined Fractional Laser Resurfacing for Deep Scalp Defect

**DOI:** 10.1002/ccr3.70677

**Published:** 2025-08-03

**Authors:** Daniel Ricardo Galimberti, Gabriella Benites Andrade, Pamela Montaño Salguero, Irene Fusco

**Affiliations:** ^1^ Coordinador de láser de Derma Internacional Centre Buenos Aires Argentina; ^2^ Derma Internacional Centre Buenos Aires Argentina; ^3^ El.En. Group Italy

**Keywords:** caucasian patient, fractional laser, granulation tissue, scalp defect, squamous cell carcinoma (SCC), wound healing

## Abstract

Among the techniques to approach scalp reconstruction, the use of combined fractional laser skin resurfacing (10,600 nm/1540 nm) is a promising therapy that results in rapid wound closure, as demonstrated in this clinical case describing a man with a deep scalp wound who was successfully treated with combined fractional laser.

## 1 Introduction

In dermatological surgery, depending on the defect, there are certain areas where it is more difficult to create a surgical flap. This is due to the lack of adjacent tissue that allows closure by first intention. For this reason, closure by secondary intention is an excellent option in these cases, obtaining good aesthetic results [1].

There are several techniques to approach scalp reconstruction, such as local flaps, free flaps, the use of skin expanders, grafts, artificial dermal substitutes, and negative pressure therapy [1]. Historically, the gold standard for reconstructive surgery has been autologous bone grafts. However, it takes a long time for enough granulation tissue to grow to perform skin grafting successfully. The freshly closed wound may be put at risk by subsequent skull reconstruction, including the reopening of soft tissue [2]. Major challenges still exist, nevertheless, because of the comparatively high rates of protrusion, resorption, and infection as well as the high risk of donor site morbidity [3, 4, 5].

It is important to know that when the calvarium is exposed without periosteum, granulation is affected and in the case of grafts used on exposed bone, they may not survive [6].

Burring of the outer table of the calvarium and immediate skin grafting is a simple and frequently used technique that leads to the healing of exposed bone/scalp defects in 90% of cases within 3 weeks [7, 8, 9].

Nevertheless, certain factors have a negative influence on the achievement of this objective, such as closure time, related to the location, size, and depth.

A fundamental part of the re‐epithelialization process is to keep the occlusive dressing moist since a short exposure to the external environment could generate desiccation and necrosis in the superficial bone layer [10, 11].

In the past, performing this procedure with a drill or chisel was common; the disadvantages were unpleasant auditory and vibratory stimulation, which led to performing it in the operating room with general anesthesia [12].

As substitutes, a number of alloplastic materials have been developed, such as calcium phosphate‐based injectable/moldable bone cement, polymethyl methacrylate, polyether ether ketone (PEEK), polyethylene, and titanium. Poor integration of soft tissues and bone is the main drawback of these materials. This can lead to implant exposure, infection, and eventually implant removal [13, 14]. Though frequently performed, cranioplasty has a high risk of complications and costs [15] necessitating the development of novel approaches.

Recently, laser technique has proven effective in this field. The complication risk of ablative laser encouraged the development of nonablative and, more recently, fractional resurfacing in an attempt to decrease risk and accelerate recovery times [16]. Fractional CO_2_ therapy has fewer side effects than nonfractional lasers, with only a few reports of scarring [17, 18].

The re‐epithelialization is supported by local keratinocytes at the wound's edges and allowed by the epithelial stem cells of hair follicles and sweat glands [19, 20]. According to evidence from the literature, fractional resurfacing with CO_2_ has been shown to positively affect the granulation process by modulating the secretory pathway of cytokines, influencing the wound healing process.

Particularly, fractional ablative CO_2_ lasers can successfully promote skin regeneration with much less downtime and adverse effects compared to conventional lasers.

Inflammatory reaction, proliferative process, and tissue remodeling are the three consecutive phases that make up the well‐defined process of wound repair following surgery [21].

Several studies have shown that fractional CO_2_ lasers are successfully used for surgical scar management [22], to enhance the function and appearance of burn scars [23, 24, 25] for atrophic postoperative and traumatic scarring treatment [26, 27] with improvements in the scars' texture and color [28] and increasing wound healing response. Topical agents are often employed following fractional CO_2_ laser treatments to facilitate recovery [29].

Additionally, already several published studies have demonstrated that the simultaneous combination of the CO_2_ wavelength (10,600 nm) and GaAs wavelength (1540 nm) represents an efficient therapeutic approach for skin rejuvenation, improving skin laxity, and for burn/postsurgical scars management [23, 30, 31, 32, 33, 34, 35].

**FIGURE 1:**
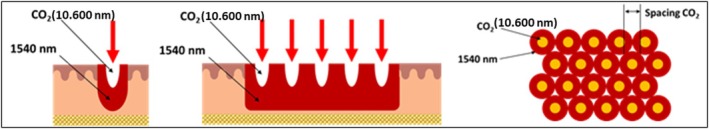
A schematic graphical representation of the action of the two wavelengths to better show the ablation and thermal effect of both laser emissions, alone or in combination. Front view (A) and top view (B). Courtesy of DEKA M.E.L.A. DEKA M.E.L.A represents the laser manufacturer.

In addition, recent published research showed that the synergistic action of the two wavelengths increases cell turnover [36, 37]. Actually, the sequential effect with the CO2 and infrared wavelengths consistently guarantees the healing times of the fractionated emission modes while extending and improving the thermal effect for more effective treatment in tissue remodeling, as shown in Figure 1.

However, depending on the intensity used, fractional laser therapy may occasionally even cause hyperpigmentation (PIH) [38].

To avoid undesirable effects such as hyperpigmentation (PIH), a specific scanning system (Scar3) was developed [39] and it was used for the treatment of a deep scalp defect in this case report.

The efficacy of this scanner unit in the neocollagenesis activation processes was already demonstrated in a recently published study [40].

On these premises, this case report describes a 75‐year‐old Hispanic Latino man with a deep scalp defect following resection of a dermal squamous cell carcinoma, successfully treated using combined fractional laser skin resurfacing (10,600 nm/1540 nm) also through the support of this laser scanner type.

## 2 Case Presentation

In this case report, a 75‐year‐old Hispanic Latino male patient, who presented a surgical site with exposed bone tissue in the upper front cranial area, was treated in the Derma Internacional center (Buenos Aires, Argentina).

The patient had a 9‐month history of a rapidly growing squamous cell carcinoma (SCC) in the upper front cranial area. After receiving Mohs surgery to remove it, the use of a skin flap caused the patient's tissue necrosis.

Consequently, the patient developed a massive lesion that exposed his cranial bone. The form of granulation in the scalp was centripetal and slow. The major axis of the lesion measured 4 cm, and the minor axis was 4 cm in diameter, with a thickness of 2 cm and a central ulceration. A 2‐mm punch biopsy confirmed the clinical diagnosis of a well‐differentiated SCC. No signs of peripheral neuropathy were present at the moment of the first clinical visit.

Based on the progression of the therapy, the doctor has prescribed antibiotics if necessary.

## 3 Methods

The Combined Fractional Laser Skin Resurfacing (10,600 nm/1540 nm) (DuoGlide system, DEKA M.E.L.A Srl, Florence, Italy) was used to treat this case of surgical site with exposed bone tissue in the upper front cranial area. DEKA M.E.L.A represents the laser manufacturer. Thanks to this technology, two wavelengths (10,600 nm and 1540 nm) can be emitted in a synergic emission mode, and two types of scanners, Scar3 and μScanDOT, can be set up. The fractionated μScanDOT scanner combines a CO_2_ (10,600 nm) and 1540 nm, while the Scar3 scanner uses the CO_2_ laser source.

The central area with exposed bone was treated with the Scar3 scanner 1 week after the appearance of the surgical lesion that leads to bone exposure, to reach deep into the bone and perform hemorrhagic stippling to stimulate the growth of the overlying tissue. The calvaria was perforated with a high peak emission mode (HP Pulse) with a power of 15 W and a distance of 500 μm. The small spot of the Scar3 scanner and the possibility of using a cold pulse (HP Pulse) thus minimize the unwanted thermal action of the laser on the tissue.

The periphery of the lesion was treated with the CO_2_ + 1540 laser combination (μScanDOT scanner) and with a smart pulse emission mode (SP pulse) of 6 W, stack3, 1000 ms dwell time, and with 1540 nm source using an energy for DOT of 30 mJ and 600 μm spacing. Dressings were kept moist to avoid bone desiccation. We used calcium alginate dressings moistened with a physiological solution.

The patient underwent 3 treatments at 1‐month treatment intervals.

## 4 Conclusion and Results (Outcome and Follow‐Up)

**FIGURE 2:**
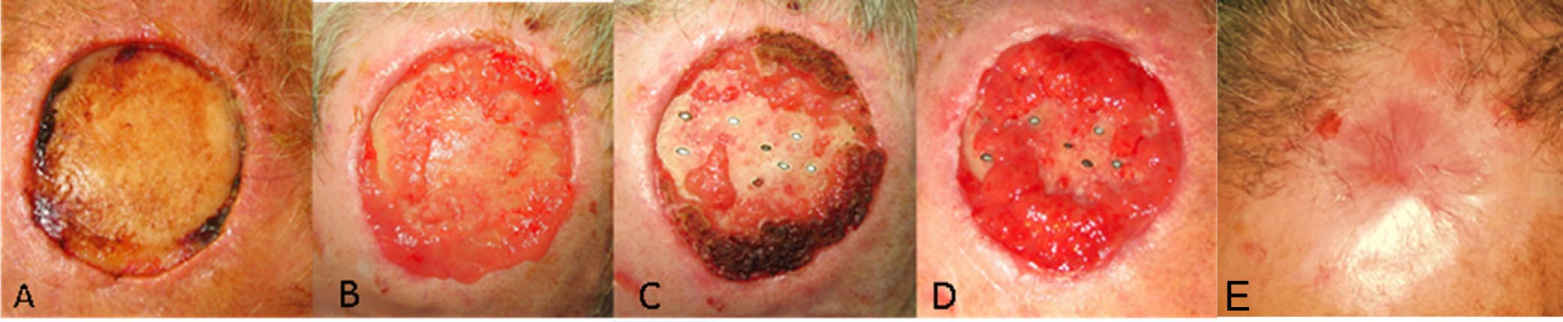
Clinical photographic documentation of patient's scalp defect following combined fractional laser therapy. The time course of the patient's wound healing at baseline (A), at 1 month after laser treatment (B), at 2 months after laser treatment (C), at 3 months after laser treatment (D), and after laser treatment at a one‐year follow‐up (E). A complete closure of the patient's wound was observed. The multiple black and white ovals in Figures C and D represent exposed bone following laser drilling, which was performed to restore blood flow.

The treatment showed excellent results with rapid wound closure 12 months from follow up, as shown in Figure 2. The patient did not report pain or discomfort after the laser procedure.

The patient signed a written form indicating their understanding of the procedure's risks.

## 5 Discussion

The purpose of the proposed technique was to access the diploe. This technique seems to be more advanced than the one used in the study by Nisticò et al. [41]. The effectiveness of a laser procedure combining an ablative CO_2_ laser (in both fractional and ablative mode) with a flash lamp pulsed dye laser (FPDL) was evaluated to remove fibrotic tissue and minimize the vascular component from a surgical nasal dorsum scar caused by flap necrosis. This is because the new technique does not only act on the edges of the wound. The first stimulation was performed with CO_2_ laser emission (with Scar3 scanner unit) in the scar central area, and then the edges of the wound were irradiated with the μScanDOT. In this way, the ablative action of CO_2_ on the edges of the wound is minimized to favor the reconstruction of the growth margins of the wound through deep nonablative stimulation.

Scar3 scanner unit can reduce the risk of post‐treatment hyper‐ and hypopigmentation with good bleeding control, thanks to its small spot size, which can generate a small microthermal zone (DOT), with greater depth of ablative action but emitting less energy, consequently avoiding the necrotic processes of the tissues. Scar3 was specifically developed for deep scar remodeling, and its use has produced excellent and good results as demonstrated by Scarcella et al. [42]

By successively transmitting one or both wavelengths (1540 nm and 10,600 nm) on the same DOT, the μScan DOT enables the delivery of novel and more effective treatments as well as a configurable balance between ablation and coagulation depths. Additionally, the entire scanning region can be heated uniformly, continuously, and noncoagulatively at the second wavelength of 1540 nm. It is feasible to penetrate deeper and deeper into the dermis (which is not pleasantly achievable with the ablative laser alone) by employing spots of 1000 μm that are emitted on the same axis as the DOT and the standard CO2 spacing parameters (500 μm) for dermatologic applications [43]. The objective of the technique that uses the dual wavelength laser was to facilitate and accelerate healing time, allowing granulation to occur from the base or center of the surgical site with exposed bone tissue.

This technique was performed on an outpatient basis, so it was optional to administer oral analgesics and/or benzodiazepines before the procedure. Complications to be monitored are bone desiccation, bleeding, intracranial infection, perforation of the dura mater, cerebrospinal injury, and cerebrospinal fluid leak.

Patient selection is essential to avoid complications. An essential contraindication is when the dura mater is exposed. Other relative contraindications are previous radiotherapy, diabetes, immunosuppression, and poor adherence to perform subsequent healing.

The main benefit of this method is that it gets greater total thermal lesion coverage in the scanning region of 10,600 nm and 1540 nm wavelengths than with CO_2_ wavelength alone. Increases in the coagulation zone cause greater tissue shrinkage but remain within a range that does not affect tissue healing. The 10,600 nm–1540 nm sequence improves the effects, both in terms of tone strengthening (due to a greater shrinkage effect) and stimulation (due to a greater volumetric thermal effect) [43]. As demonstrated in the study by Nisticò et al. [43] with the help of 1540 nm, this coagulative extension effect under the healthy epidermis, or between the two successive CO_2_ DOTs, produces a more uniform remodeling and mimics the results of conventional resurfacing but now has a healing time comparable to fractional CO_2_ alone. Our findings support research by Mezzana et al. [35] and Snast and collaborators [44], which found that using two wavelengths simultaneously (10,600 nm and near‐infrared) did not alter CO_2_ laser ablation but did increase the coagulation zone, increasing treatment effectiveness and safety while decreasing downtime. This technique can be recommended if there is a defect in the scalp without the possibility of reconstruction. Indeed, if the shell without periosteum is intact, the possibilities of granulation are greater. Consequently, the granulation process is slow and generates a disadvantage because it increases the risks of infections and long‐term hospitalization. The advantage when performing fenestration with the CO_2_ laser is providing thermal stimulation in the perilesional tissue with the fractional mode to generate a centripetal closing. However, the precision of the points, the controlled thermal damage, and the possibility of performing it on an outpatient basis with local anesthesia represent additional benefits of the proposed technique.

Additionally, the combined application of the two wavelengths indicated a greater contraction effect on ex vivo skin.

Finally, compared to the other studies cited in the Introduction section [27, 29] our research studied the effects of laser therapy up to 1 year of follow‐up, monitoring the progression of wound healing at different follow‐up times (at 1, 2 and 3 months after laser treatment).

Our results demonstrated that the proposed combined fractional laser skin resurfacing technique led to a rapid wound closure of a deep scalp defect following surgical resection of a dermal squamous cell carcinoma, representing an appropriate and promising therapy for wound healing and granulation stimulation for scalp reconstruction. Further clinical cases will be necessary to validate the effectiveness of this laser technique for scalp reconstruction.

## Author Contributions

**Daniel Ricardo Galimberti:** conceptualization, data curation, formal analysis, funding acquisition, investigation, methodology, project administration, resources, software, supervision, validation, visualization, writing – original draft, writing – review and editing. **Gabriella Benites Andrade:** conceptualization, data curation, formal analysis, funding acquisition, investigation, methodology, project administration, resources, software, supervision, validation, visualization, writing – review and editing. **Pamela Montaño Salguero:** conceptualization, data curation, formal analysis, funding acquisition, investigation, methodology, project administration, resources, software, supervision, validation, visualization, writing – review and editing. **Irene Fusco:** supervision, validation, visualization, writing – original draft, writing – review and editing.

## Disclosure

Institutional Review Board Statement: No activity was carried out outside the scope of the device intended purpose.

## Consent

A written informed consent was obtained from the patient to publish the case report.

## Conflicts of Interest

I.F. is employed at El.En. Group. The remaining authors have no conflicts of interest to declare.

## Data Availability

Data that support the study findings are available on request from the corresponding author (I.F).
